# Risk factors for radiation pneumonitis in lung cancer patients with subclinical interstitial lung disease after thoracic radiation therapy

**DOI:** 10.1186/s13014-021-01798-2

**Published:** 2021-04-13

**Authors:** Fangjuan Li, Hui Liu, Hongyu Wu, Shixiong Liang, Yaping Xu

**Affiliations:** 1 Department of Radiation Oncology, Shanghai Pulmonary Hospital, Tongji University School of Medicine, No. 507 Zhengmin Road, Yangpu District, Shanghai, 200433 People’s Republic of China; 2 Department of Radiation Oncology, Affiliated Cancer Hospital of Guangxi Medical University, Cancer Institute of Guangxi Zhuang Autonomous Region, Nanning, Guangxi People’s Republic of China

**Keywords:** Lung cancer, RP, ILD

## Abstract

**Background:**

Previous studies have found that patients with subclinical interstitial lung disease (ILD) are highly susceptible to developing radiation pneumonitis (RP) after thoracic radiation therapy. In the present study we aimed to evaluate the incidence of and risk factors for RP after thoracic intensity-modulated radiation therapy in lung cancer patients with subclinical ILD.

**Methods:**

We retrospectively analyzed data from lung cancer patients with subclinical ILD who were treated with thoracic intensity-modulated radiation therapy with a prescribed dose of ≥ 50 Gy in our institution between January 2016 and December 2017.

**Results:**

Eighty-seven consecutive lung cancer patients with subclinical ILD were selected for the study. The median follow-up period was 14.0 months. The cumulative incidence of grades ≥ 2 and ≥ 3 RP at one year was 51.0% and 20.9%, respectively. In the multivariate analysis, a mean lung dose ≥ 12 Gy was a significant risk factor for grade ≥ 2 RP (*p* = 0.049). Chemotherapy with gemcitabine in the past, V5 ≥ 50%, and subclinical ILD involving ≥ 25% of the lung volume were significantly associated with grade ≥ 3 RP (*p* = 0.046, *p* = 0.040, and *p* = 0.024, respectively).

**Conclusion:**

Mean lung dose is a significant risk factor for grade ≥ 2 RP. Lung cancer patients who have received chemotherapy with gemcitabine in the past, V5 ≥ 50%, and those with subclinical ILD involving ≥ 25% of lung volume have an increased risk of grade ≥ 3 RP in lung cancer patients with subclinical ILD.

## Introduction

Radiation pneumonitis (RP) is a common complication of radiotherapy for lung cancers. The incidence of symptomatic RP is approximately 15–40% [1]. Dose-volume histogram-based dosimetric parameters, including the percentage of lung volume receiving a dose of ≥ 20 Gy (V20), the mean lung dose (MLD), treatment factors such as sequential or concurrent chemotherapy schedules, tumor factors such as tumor size and location, and patient factors including poor pulmonary function and concomitant disease are predictive markers for RP [2–9].

Subclinical interstitial lung disease (ILD) has a higher incidence in patients with lung cancer than in the general population [10]. Previous studies found that patients with subclinical ILD were more susceptible to developing RP after thoracic radiation therapy (TRT) [10–16].

Few studies have investigated the correlation between subclinical ILD and RP. There have been no reports on the incidence of and risk factors for RP in lung cancer patients with subclinical ILD after intensity-modulated radiation therapy (IMRT). This single-institution study was conducted to determine the incidence of and risk factors for RP after IMRT in lung cancer patients with subclinical ILD.

## Materials and methods

### Patients

Lung cancer patients with subclinical ILD who were treated with thoracic IMRT in our institution between January 2016 and December 2017 were retrospectively analyzed. The inclusion criteria were as follows: (1) patients diagnosed with lung cancer by histology or cytology; (2) interstitial lung changes on high-resolution Computed Tomography (HRCT) images before radiotherapy and chemotherapy; (3) Karnofsky performance status scale ≥ 70 and ability to endure IMRT at a total dose of the equivalent dose in 2.0 Gy/(fraction per day) (EQD2) ≥ 50 Gy; (4) age ≥ 18 years; (5) no other serious medical conditions; (6) RT with concurrent or sequential chemotherapy; (7) no TRT received previously; (8) thoracic CT images available for evaluation before and after TRT; (9) follow-up time of more than six months for patients without RP; and (10) patients were inoperable. Individuals with TRT terminated due to non-radiotherapy-related complications for more than seven days were excluded.

### Radiotherapy

All patients underwent a planning CT scan when immobilized in a supine position with their arms raised in a customized vacuum-lock mold. Simulation CT images were taken at 0.5 cm increments over the region of interest. Treatment planning was performed using an ADAC Pinnacle™ (Philips Medical Systems) system. Treatment consisted of 6 or 10 MV photon thoracic IMRT using a Siemens Artiste (Oncology Care Systems, Siemens Medical Solutions, CA, USA) digital linear accelerator. The target volumes were set manually by experienced radiation oncologists focused on lung cancer.

The gross tumor volume (GTV) was defined as the volume of a primary tumor demonstrated by a CT scan and metastatic lymph nodes based on pretreatment chest CT scan and/or [18F]-fluorodeoxyglucose positron emission tomography/CT images, bronchoscopy or other approaches. The clinical target volume (CTV) was typically a 0.6–0.8 cm expansion of the GTV, including the primary tumor in the lung and the drainage area of metastatic lymph nodes. The CTV of prophylactic postoperative radiotherapy was determined based on postoperative pathology, including the bronchial stump, ipsilateral hilar, and drainage area of tumor-positive lymph nodes. The planning target volume (PTV) was defined by adding margins at the discretion of radiation oncologists. The margins were typically 0.5–1.0 cm, depending on respiratory motion and patient fixation. The goal of the therapy was to deliver the prescribed dose to at least 95% of the PTV, while meeting normal tissue constraints. The total dose was ≥ 50.0 Gy, and was generally delivered at 2.0–3.0 Gy/(fraction per day), for five fractions per week. If the lung dose exceeded the safety range, the total dose was reduced as appropriate. Informed consent was obtained from all patients prior to radiotherapy. Ethical approval was obtained from the Ethical Review Committee of Shanghai Pulmonary Hospital, Tongji University School of Medicine, China.

### Chemotherapy

The concurrent chemotherapy regimen consisted of platinum combined with pemetrexed, paclitaxel, docetaxel, vinorelbine, or etoposide. Patients who were older, had reached stage IV, had poor pulmonary function, anemia, abnormal liver or renal function, or who exhibited progression after first-line chemotherapy were given single-agent concurrent chemotherapy or sequential chemoradiotherapy. Chemotherapy was generally performed in four to six cycles every three to four weeks.

### Diagnosis of subclinical ILD

Data from lung cancer patients with subclinical ILD detected using HRCT images who were treated with thoracic IMRT in our institution between January 2016 and December 2017 were analyzed. The diagnosis of subclinical ILD was based on pretreatment HRCT images with an axial slice thickness of 0.1 cm in a lung window. Reticular abnormalities, traction bronchiectasis, bilateral independent ground-glass abnormalities, honeycombing, and nonemphysematous cysts were considered to be indicative of subclinical ILD [17–19]. The diagnosis of subclinical ILD and the CT scans were evaluated independently by a radiologist and two physicians specializing in pulmonology.

### Follow-up

Patients were re-evaluated at one to two months post treatment and subsequently every three months. The endpoint was the incidence of grade ≥ 2 RP. Adverse events were graded using the Common Terminology Criteria for Adverse Events Version 4.0.

### Statistical analysis

Correlations between RP and the risk factors were analyzed using Chi-square tests, Student’s t-tests, or Mann–Whitney U tests for univariate analysis. Receiver operating characteristic curves were generated to determine the optimal cut-off value of continuous variables. Logistic regression analysis was performed to evaluate the correlations between RP and the risk factors using multivariate analysis. The cumulative incidence of RP was estimated using the Kaplan–Meier method, and differences between the groups were assessed using log-rank tests. The overall survival (OS) was defined as the interval between the date of diagnosis and the date of death or last follow-up. OS was also estimated using the Kaplan–Meier method. Statistical analyses were performed using IBM SPSS software 22.0 for Mac. A* p* value less than 0.05 was considered to be statistically significant.

## Results

From January 2016 to December 2017, a total of 87 consecutive lung cancer patients with subclinical ILD, aged 48 to 86 years with a median age of 67 years, were enrolled in the study. The tumor stage was determined according to the 8th edition of the Union for International Cancer Control TNM staging system for lung cancer. None of the patients had been diagnosed with ILD clinically or via lung biopsy prior to receiving chemotherapy or radiotherapy. None of the patients had received treatment with any of the currently available medicines for ILD. Seventy-six patients had received chemotherapy prior to radiotherapy. The median duration of chemotherapy was two cycles (range, 0–6 cycles). Two patients had previously received epidermal growth factor receptor tyrosine kinase inhibitor. The characteristics of the patients are shown in Table 1.

**Table 1:** Characteristics of the patients

Factors	N (%)
Gender	
Male	81 (93.1)
Female	6 (6.9)
Age (years)	
< 70	57 (65.5)
≥ 70	30 (34.5)
Pathological types	
NSCLC	57 (65.5)
Adenocarcinoma	17 (19.5)
Squamous cell carcinoma	24 (27.6)
Large cell carcinoma	1 (1.1)
Unclassified NSCLC	15 (17.2)
SCLC	30 (34.5)
Tumor stage	
I	2 (2.3)
IIIA	24 (27.6)
IIIB	37 (42.5)
IIIC	3 (3.4)
IV	10 (11.5)
Postoperative	11 (12.6)
Chemotherapy	
Concurrent	19 (21.8)
Sequential	68 (78.2)

NSCLC, non-small-cell lung cancer; SCLC, small-cell lung cancer

The median follow-up time was 14.0 months (range, 1.2–58.9 months). RP was observed in 19 (21.8%), 27 (31.0%), 10 (11.5%), three (3.4%), and five (5.7%) patients with grades 1, 2, 3, 4, and 5 RP, respectively. Radiotherapy was discontinued in eight patients because a grade ≥ 2 RP occurred during radiotherapy. The characteristics of the patients for whom radiotherapy was discontinued are shown in Table 2. Five patients developed grade 5 RP; their characteristics are shown in Table 3.

**Table 2:** Characteristics of patients who discontinued radiotherapy

No	Age (years)/Gender	Smoking history (pack-years)	Pathological type/Tumor stage	Treatment modality	Induction chemotherapy regimen	Radiotherapy dose (Gy/fraction)	Percentage of lung volume affected in subclinical ILD (%)	Grade of RP
1	68/Male	40	Squamous cell carcinoma/IIIB	RT	Cisplatin + gemcitabine	44.0/22	≥ 25	4
2	77/Male	50	Squamous cell carcinoma/IIIB	RT	Carboplatin + gemcitabine	49.5/22	≥ 25	5
3	67/Male	No	Squamous cell carcinoma/postoperative stump recurrence	RT	Cisplatin + gemcitabine	24.0/12	< 25	2
4	63/Male	80	Adenocarcinoma/IIIB	RT	Cisplatin + gemcitabine	58.0/29	< 25	3
5	65/Male	40	NSCLC/IIIB	RT	Cisplatin + vinorelbine	38.0/19	< 25	4
6	69/Male	50	Squamous cell carcinoma/IIIB	RT	Carboplatin + gemcitabine	38.0/19	≥ 25	5
7	52/Male	60	Adenocarcinoma/IIIB	RT	Cisplatin + pemetrexed	30.0/15	< 25	2
8	67/Male	No	SCLC/IIIB	RT	Carboplatin + etoposide	40.0/20	< 25	2

RP, radiation pneumonitis; ILD, interstitial lung disease; RT, radiation therapy; NSCLC, non-small-cell lung cancer; SCLC, small-cell lung cancer

**Table 3:** Characteristics of patients with grade 5 RP

No	Age (years)/Gender	Smoking history (pack-years)	Pathological types/Tumor stage	Treatment modality	Concurrent chemotherapy regimen	Induction chemotherapy regimen	Radiotherapy dose (Gy/fraction)	MLD (Gy)	Percentage of lung volume affected in subclinical ILD (%)	Time from treatment to grade 5 RP (months)
1	59/Male	20	Adenocarcinoma/postoperative recurrence	CCRT	Cisplatin + pemetrexed	Cisplatin + gemcitabine	60.0/30	12.12	< 25	3.3
2	77/Male	50	Squamous cell carcinoma/IIIB	RT	None	Carboplatin + gemcitabine	49.5/22	13.89	≥ 25	4.1
3	69/Male	50	Squamous cell carcinoma/IIIB	RT	None	Carboplatin + gemcitabine	38.0/19	12.19	≥ 25	1.2
4	71/Male	60	SCLC/IIIA	CCRT	Cisplatin + etoposide	Cisplatin + etoposide	60.0/30	12.65	< 25	6.8
5	62/Male	30	Squamous cell carcinoma/postoperative recurrence	CCRT	Carboplatin + paclitaxel	None	60.0/30	11.92	< 25	3.0

RP, radiation pneumonitis; MLD, mean lung dose; ILD, interstitial lung disease; RT, radiation therapy; CCRT, concurrent chemoradiotherapy; SCLC, small-cell lung cancer

**Fig. 1:**
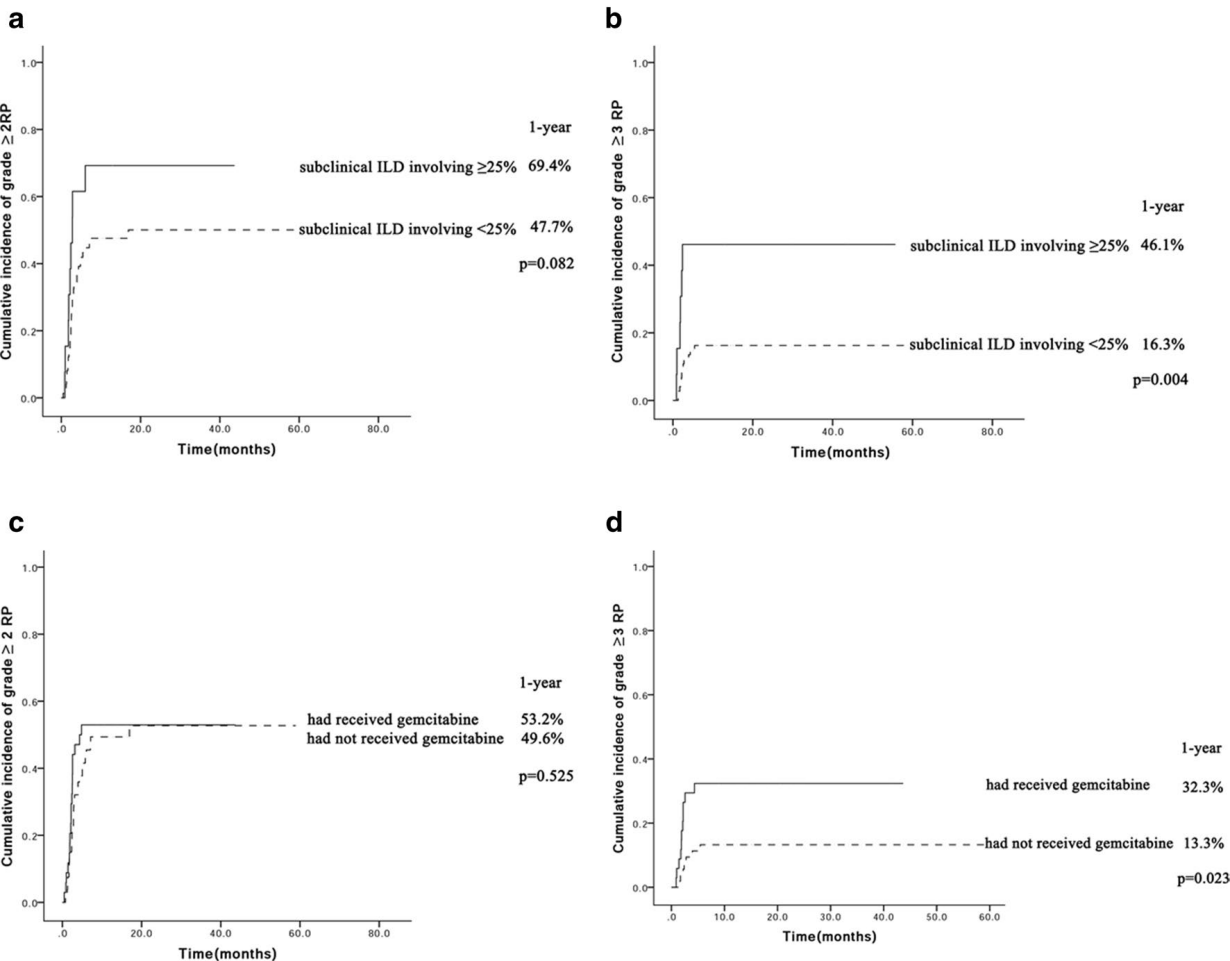
**a** Cumulative incidence of grade ≥ 2 RP in patients with different involvement volumes of subclinical ILD; **b** Cumulative incidence of grade ≥ 3 RP in patients with different involvement volumes of subclinical ILD; **c** Cumulative incidence of grade ≥ 2 RP in patients who had (solid line) or had not (dashed line) received chemotherapy with gemcitabine in the past; **d** Cumulative incidence of grade ≥ 3 RP in patients who had (solid line) or had not (dashed line) received chemotherapy with gemcitabine in the past

The cumulative incidence of grades ≥ 2 RP at one year was 51.0% and that of grades ≥ 3 RP was 20.9%. Although the percentage of lung volume affected in subclinical ILD did not significantly increase the cumulative incidence of grade ≥ 2 RP (69.4%vs.47.7%, *p* = 0.082, Fig. 1a), the cumulative incidence of grade ≥ 3 RP was significantly higher in patients with subclinical ILD involving ≥ 25% of lung volume than those with < 25% involvement of lung volume (46.1% vs. 16.3%, *p* = 0.004, Fig. 1b). Gemcitabine chemotherapy before radiotherapy did not significantly affect the cumulative incidence of grade ≥ 2 RP (53.2% vs. 49.6%, *p* = 0.525, Fig. 1c), but the cumulative incidence of grade ≥ 3 RP was significantly higher in patients who had received chemotherapy with gemcitabine in the past than in those who had not (32.3% vs. 13.3%, *p* = 0.023, Fig. 1d).

Table 4 shows the correlations between the risk factors and RP. In the univariate analysis, tumor location (upper lobe vs. middle or lower lobe) and MLD were significantly associated with grade ≥ 2 RP (*p* = 0.043 and *p* = 0.024, respectively). The risk of grade ≥ 3 RP was higher in patients who had received chemotherapy with gemcitabine in the past, and in those who had subclinical ILD involving ≥ 25% of lung volume (*p* = 0.031 and *p* = 0.037, respectively).

**Table 4:** Correlation between factors and RP by univariate analysis

Factors	Grade ≥ 2 RP (N = 45)			Grade ≥ 3 RP (N = 18)		
	Grade < 2 RP	Grade ≥ 2 RP	*p* value	Grade < 3 RP	Grade ≥ 3 RP	*p* value
Gender			0.175			0.439
Male	37	44		63	18	
Female	5	1		6	0	
Age (years)			0.256			0.659
< 70	25	32		46	11	
≥ 70	17	13		23	7	
Smoking history			0.165			0.493
No	15	10		21	4	
Yes	27	35		48	14	
Pathological type			0.503			0.219
NSCLC	29	28		43	14	
SCLC	13	17		26	4	
Tumor stage			0.092			0.692
I	2	0		2	0	
III	28	36		51	13	
IV	4	6		7	3	
Postoperative	8	3		9	2	
Tumor location			0.043			0.120
Upper lobe	32	25		48	9	
Middle or lower lobe	10	20		21	9	
Chemotherapy with gemcitabine in the past			0.856			0.031
No	26	27		46	7	
Yes	16	18		23	11	
Concurrent chemotherapy			0.142			0.782
No	30	38		53	15	
Yes	12	7		16	3	
Distribution of subclinical ILD			0.275			0.157
Lateral	7	4		11	0	
Bilateral	35	41		58	18	
Morphology of subclinical ILD			0.399			0.137
No honeycombing	34	33		56	11	
Honeycombing	8	12		13	7	
Percentage of lung volume affected in subclinical ILD			0.171			0.037
< 25%	38	36		62	12	
≥ 25%	4	9		7	6	
Pulmonary emphysema			0.658			0.775
No	6	8		12	2	
Yes	36	37		57	16	
Total dose (Gy)			0.834			0.349
EQD2 < 60.0	13	13		19	7	
EQD2 ≥ 60.0	29	32		50	11	
Single fraction dose			0.826			1.000
2.0 Gy	38	39		61	16	
> 2.0 Gy, ≤ 3.0 Gy	4	6		8	2	
FVC%	89.53 ± 14.50	92.15 ± 25.83	0.632	89.93 ± 22.58	94.53 ± 17.75	0.472
FEV1/ FVC (%)	74.71 ± 9.05	74.05 ± 9.89	0.782	74.57 ± 8.52	73.56 ± 12.40	0.720
MLD (Gy)	11.52 ± 3.36	12.97 ± 2.45	0.024	12.00 ± 3.15	13.29 ± 2.11	0.106
V5 (%)	46.07 ± 12.51	48.24 ± 9.54	0.363	46.19 ± 11.69	51.06 ± 7.25	0.097
V10 (%)	33.14 ± 9.84	35.00 ± 7.20	0.316	33.68 ± 9.26	35.72 ± 5.04	0.216
V20 (%)	20.62 ± 6.88	21.89 ± 4.46	0.314	21.10 ± 6.14	21.94 ± 4.05	0.583
V30 (%)	14.24 ± 5.64	16.07 ± 4.08	0.085	14.91 ± 5.12	16.22 ± 4.22	0.321
GTV (cc)	97.80(3.75–398.99)	120.17(20.47–497.86)	0.288	105.40(3.75–398.99)	105.14(20.47–497.86)	0.991
PTV (cc)	355.88(69.26–2601.29)	392.60(300.77–1070.38)	0.139	379.12(69.26–2601.29)	349.95(257.32–1070.38)	0.608

RP, radiation pneumonitis; NSCLC, non-small-cell lung cancer; SCLC, small-cell lung cancer; ILD, interstitial lung disease; FVC, forced vital capacity; FEV1, forced expiratory volume in one second; FVC%, percentage forced vital capacity; EQD2, equivalent dose in 2.0 Gy/(fraction per day); MLD, mean lung dose; V5, percentage of lung volume receiving ≥ 5 Gy; V10, percentage of lung volume receiving ≥ 10 Gy; V20, percentage of lung volume receiving ≥ 20 Gy; V30, percentage of lung volume receiving ≥ 30 Gy; GTV, gross tumor volume; PTV, planning target volume

In the multivariate analysis, MLD ≥ 12.0 Gy was a significant risk factor for grade ≥ 2 RP (*p* = 0.049). Having received chemotherapy with gemcitabine in the past, having a V5 ≥ 50%, and having subclinical ILD involving ≥ 25% of the lung volume were significantly associated with grade ≥ 3 RP (*p* = 0.046, *p* = 0.040, and *p* = 0.024, respectively). The results of a binary logistic regression analysis for RP are shown in Table 5.

**Table 5:** Correlation between risk factors and RP using binary logistic regression analysis

Factors	Grade ≥ 2 RP			Grade ≥ 3 RP		
	Odds ratio (OR)	95% CI	*p* value	OR	95% CI	*p* value
MLD (≥ 12.0 Gy vs. < 12.0 Gy)	2.480	1.006–6.113	0.049	–	–	–
Tumor located in lower lobe	2.311	0.898–5.943	0.082	–	–	–
Chemotherapy with gemcitabine in the past	–	–	–	3.209	1.018–10.113	0.046
V5 (≥ 50%vs. < 50%)	–	–	–	3.429	1.056–11.140	0.040
Percentage of lung volume affected in subclinical ILD ≥ 25%	–	–	–	4.861	1.237–19.104	0.024

RP, radiation pneumonitis; MLD, mean lung dose; V5, percentage of lung volume receiving ≥ 5 Gy; ILD, interstitial lung disease; CI, confidence interval

**Fig. 2:**
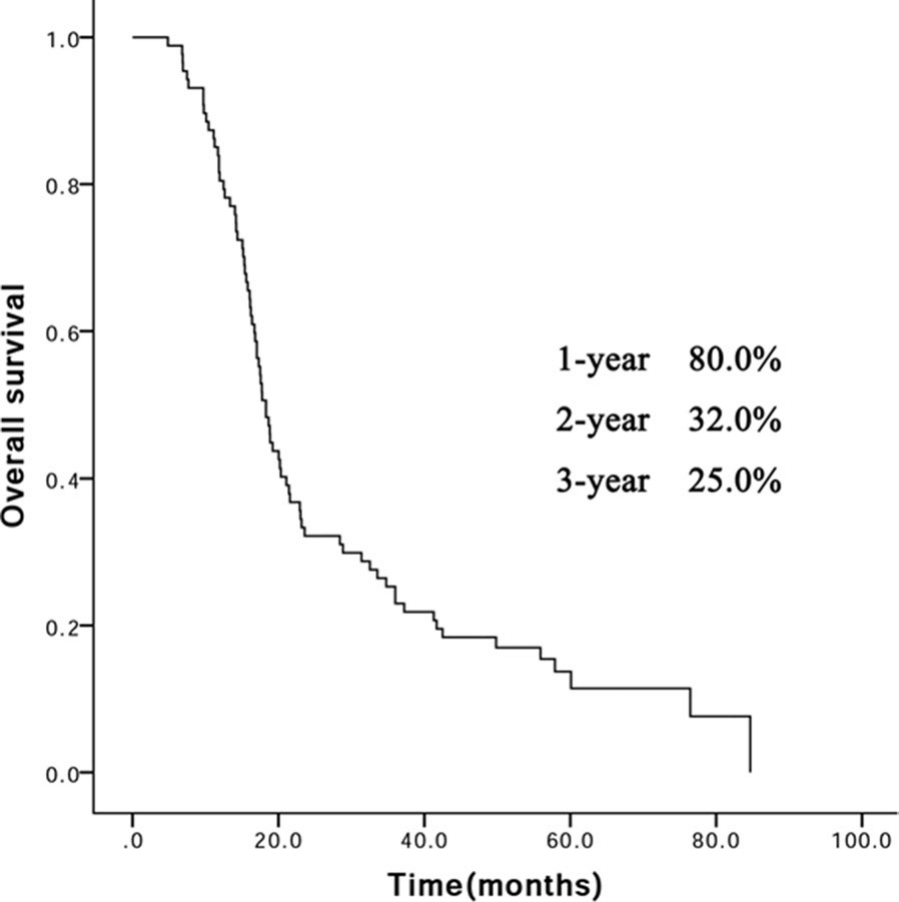
Survival curve for all patients

The median survival time for all patients was 18.3 months (range, 4.8–84.7 months). Seventy-seven patients had died at the time of writing. The one-year, two-year, three-year OS rates were 80.0%, 32.0%, and 25.0%, respectively (Fig. 2).

## Discussions

Previous studies have identified a 4%–11% prevalence of subclinical ILD in high-risk populations undergoing CT screening for lung cancer [20]. A few studies found that the incidence of grade ≥ 2 RP and grade ≥ 3 RP was significantly higher in patients with subclinical ILD than in those without subclinical ILD after stereotactic body radiotherapy (SBRT) [11, 20]. Yamaguchi and colleagues [21] reported that although subclinical ILD showed no significant correlation with grade ≥ 2 RP, three patients with extensive bilateral RP had subclinical ILD prior to receiving radiotherapy. Several studies have also found a significantly higher incidence of RP after three-dimensional conformal radiotherapy (3D-CRT) in lung cancer patients with subclinical ILD [10, 12]. Two of three patients with grade 5 RP had subclinical ILD in multiple lobes [10].

Data on the correlation between subclinical ILD and RP in patients after IMRT are limited. Our previous study revealed that subclinical ILD was a risk factor for grade ≥ 3 RP in patients with small-cell lung cancer after TRT [13]. In this single-institution retrospectively study, the cumulative incidence of grade ≥ 2 RP was 51.7%, and that of grade ≥ 3 RP was 20.7% in lung cancer patients with subclinical ILD after IMRT. The incidence of grade ≥ 2 RP in this study was consistent with that reported by previous studies, and the rate of grade ≥ 3 RP in this study was lower than that reported previously for patients with subclinical ILD following 3D-CRT [10, 12]. Sanuki et al. [22] found the rate of grade ≥ 3 RP increased from 3 to 26% in patients with subclinical ILD. Niska JR and colleagues [23] presented two cases of fatal RP in patients with limited subclinical ILD. Individuals with subclinical ILD were at higher risk of RP [16]. Recently, a study reported that proton therapy might be helpful for reducing acute and fatal complications in non-small-cell lung cancer (NSCLC) patients with idiopathic pulmonary fibrosis [24].

Although an association between preexisting subclinical ILD and RP has been reported, little is known about the relationship between RP and the CT radiological features of subclinical ILD. Some studies have graded subclinical ILD, in order to evaluate its severity. However, there is no consensus on the definition of subclinical ILD grading. Washko’s scoring criteria are commonly used [10, 20, 21, 25, 26]. Glick et al. [20] reported that Washko’s score is associated with grade ≥ 2 RP using a univariate analysis; however, there was no statistical difference identified using a multivariate analysis. Another study found that cases that exhibited honeycombing had a high potential for fatality due to severe RP after SBRT [27]. In this study, we explored the correlations between the distribution, morphology, and percentage of lung volume affected in subclinical ILD, and RP. Only the percentage of lung volume affected in subclinical ILD when it was ≥ 25% was significantly associated with the risk of grade ≥ 3 RP. It is easier to evaluate the severity of subclinical ILD by indirectly measuring the percentage of lung volume affected based on pretreatment HRCT images than by using the scoring criteria, which makes it easier to identify individuals who have a high risk of RP.

Dosimetric parameters are closely correlated to the incidence of RP. We found that MLD is a significant risk factor for grade ≥ 2 RP, and patients with a V5 ≥ 50% have an increased risk of grade ≥ 3 RP. Previous studies have also found that MLD was a predictor of RP in patients with subclinical ILD after SBRT and 3D-CRT [12, 20, 21]. A retrospective analysis reported that V5 was significantly associated with the occurrence of RP grade progression after carbon-ion radiotherapy for NSCLC with ILD [28]. Onishi et al. [29] found that a V20 ≥ 10% is a major risk factor for severe RP in stage I NSCLC patients with subclinical ILD. In the present study, no correlation was observed between the incidence of RP and V20. This lack of correlation may have arisen because we strictly controlled the limits of V20. Other studies have also failed to find a correlation between dosimetric parameters and RP in patients with subclinical ILD after 3D-CRT, SBRT and IMRT [10, 13, 30].

Gemcitabine is a first-line chemotherapy drug commonly used in advanced NSCLC. Some studies have reported that gemcitabine produces pulmonary toxicity. The use of concurrent radiotherapy and gemcitabine after induction with gemcitabine and carboplatin significantly increased the incidence of grade ≥ 3 RP up to 31.6% [3]. In 2010, the Quantitative Analyses of Normal Tissue Effects in the Clinic group indicated that gemcitabine is associated with a higher risk of pulmonary toxicity when used concurrently with thoracic RT [2]. Leprieur and colleagues [7] found that induction chemotherapy with gemcitabine before radiotherapy was associated with a high incidence of RP.

There have been no studies to date that evaluated the safety of chemotherapy with gemcitabine before TRT in patients with ILD or subclinical ILD. In the current study, chemotherapy with gemcitabine before radiotherapy was a significant factor influencing the occurrence of grade ≥ 3 RP in lung cancer patients with subclinical ILD. Three of the five patients with grade 5 RP received chemotherapy with gemcitabine before TRT, as did five of the eight patients in whom radiotherapy was discontinued due to the occurrence of grade ≥ 2 RP. One phase II clinical trial concluded that induction with gemcitabine/carboplatin followed by concurrent paclitaxel/carboplatin with conformal radiation is safe and tolerable [31]. However, this study did not assess whether patients had concomitant pulmonary diseases prior to radiotherapy.

The association between lung cancer and ILD can be explained by shared risk factors such as smoking, and physiopathology caused by fibrogenesis or cancerogenesis [32]. Decision making pertaining to treatment for lung cancer patients with ILD is difficult. The prognosis of these patients appears to be poorer than for those with lung cancer alone [32]. Treatments for lung cancer patients, including resection, chemotherapy, and radiotherapy may trigger severe pulmonary toxicities, such as acute exacerbation or RP [33]. Sato et al. retrospectively analyzed 1763 NSCLC patients with a clinical diagnosis of ILD, and demonstrated 9.3% acute exacerbation and 43.9% 30-day mortality after surgical resection [34]. Acute exacerbation of ILD sometimes occurs after chemotherapy for lung cancer [33]. Previous studies have reported that patients with subclinical ILD are more susceptible to developing severe, extensive RP after TRT in lung tumor [10–16, 21, 23]. The classical RP changes in the lungs have been considered to be confined to the site of irradiation. However, in the early literature, there have been several reports of extensive RP occurring beyond the irradiated field [25, 33, 35]. Although there is currently no clear understanding of the pathophysiology underlying the increased rate and severity of RP in ILD patients, a hypothesis of lymphocyte-mediated hypersensitivity reaction induced by radiation therapy has been postulated [25, 33, 35]. Studies into radiotherapy that triggers severe pulmonary toxicities in these patients are scarce. More data are needed to clarify the mechanisms involved in this phenomenon.

In conclusion, MLD is a significant risk factor for grade ≥ 2 RP, and lung cancer patients who have received chemotherapy with gemcitabine in the past, who have V5 ≥ 50%, and those with subclinical ILD involving ≥ 25% of lung volume have an increased risk of grade ≥ 3 RP. The dose-volume parameters should be strictly controlled to ensure the safety of radiotherapy. It is recommended that chemotherapy with gemcitabine be avoided prior to radiotherapy in lung cancer patients with subclinical ILD. Radiation oncologists should carefully select treatments for lung cancer patients with subclinical ILD by considering the clinical characteristics and the IMRT-induced benefits and toxicities.

This study had some limitations. As a single-center retrospective study, with a small sample size and short inclusion time, this study may be affected by selection bias and confounding factors. It was difficult to accurately distinguish RP from other types of pneumonitis, because RP is a clinical diagnosis, and can be confounded by preexisting or comorbid disease, including subclinical ILD exacerbations, tumor progression, or infection. The diagnosis of subclinical ILD was based on pretreatment HRCT imaging and was evaluated by a radiologist and two physicians specializing in pulmonology, so subjective judgments made by these individuals may differ. A larger, prospective multi-center study is needed to confirm the conclusions drawn from the data gathered in this study.

## Data Availability

Support data is available to interested readers upon reasonable request to corresponding author.

## Abbreviations

CCRT: Concurrent chemoradiotherapy
CI: Confidence interval
CTV: Clinical target volume
EQD2: Equivalent dose in 2.0 Gy/(fraction per day)
FEV1: Forced expiratory volume in one second
FVC: Forced vital capacity
FVC%: Percentage forced vital capacity
GTV: Gross tumor volume
HRCT: High-resolution Computed Tomography
ILD: Interstitial lung disease
IMRT: Intensity-modulated radiation therapy
MLD: Mean lung dose
NSCLC: Non-small-cell lung cancer
OS: Overall survival
PTV: Planning target volume
RP: Radiation pneumonitis
RT: Radiation therapy
SBRT: Stereotactic body radiotherapy
SCLC: Small-cell lung cancer
TRT: Thoracic radiation therapy
V5: Percentage of lung volume receiving ≥ 5 Gy
V10: Percentage of lung volume receiving ≥ 10 Gy
V20: Percentage of lung volume receiving ≥ 20 Gy
V30: Percentage of lung volume receiving ≥ 30 Gy
3D-CRT: Three-dimensional conformal radiotherapy
